# A new β-cyclodextrin-based nickel as green and water-soluble supramolecular catalysts for aqueous Suzuki reaction

**DOI:** 10.1038/s41598-023-48603-6

**Published:** 2023-12-02

**Authors:** Sara Payamifar, Ahmad Poursattar Marjani

**Affiliations:** Department of Organic Chemistry, Faculty of Chemistry, Urmia University, Urmia, Iran

**Keywords:** Catalysis, Green chemistry

## Abstract

A water-soluble nickel complex based on amino-β-CD was developed using a facile method and exhibits excellent catalytic performance in the Suzuki reaction in water. This synthesized complex has been characterized using UV–Vis, AAS, TGA, and FT-IR techniques. The easily synthesized novel supramolecular catalysts have been applied as a green and eco-friendly catalyst in the Suzuki coupling for preparing diverse biaryls. This result indicates that using 2.5 mol% of nickel, K_2_CO_3_ as the best base, and water as the green solvent are the best reaction conditions. This new catalyst features easy handling, low-cost, mild, and simple protocol. The use of low-cost and accessibility of the reagents, modest conditions, and good yields of products are notable characteristics of this method. Using aqueous media with this catalyst as a proper catalyst makes the presented process a fascinating method compared to most reports. Under mild reaction conditions, this green Ni(II)-β-CD catalyst displayed recyclable behavior seven times with minor loss in its catalytic activity.

## Introduction

**Figure 1:**
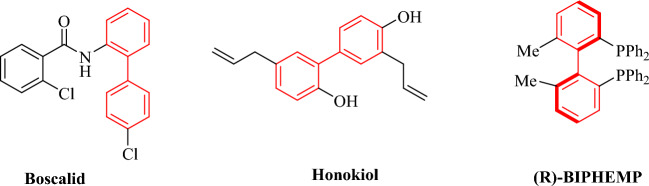
Compounds containing biaryl fragments with applications.

Bond formation is essential in many chemical reactions for synthesizing different beneficial compounds. To form C(sp^2^)–C(sp^2^) bonds, the Suzuki reaction is one of the considerably significant and influential ways. This coupling reaction occurs in aryl halides reagent with organoboranes and has been used to prepare different biologically active compounds, particularly biphenyls. The organoboron compounds are environmentally safe, low toxicity, low cost, readily accessible, and durable in moisture^1–12^. The resulting biaryls are one of the most valuable units in various compounds, including natural products, synthetic bioactive compounds, and pharmaceuticals (Fig. 1). The researcher has maintained an interest in developing different methods to form diverse biaryl compounds^13–17^.

Palladium catalysts are generally the most routine choices for various cross-coupling reactions. Some main problems in Palladium catalysts include the high price and toxicity of palladium metal and its compounds, which can limit their industrial application. Based on the principles of green chemistry, the search for low-cost transition metal catalysts to replace palladium is necessary to prepare the biaryl derivatives and continues to draw notable consideration. Recently, great concentration has been expended on using nickel as a popular option for substitute Pd in cross-coupling reactions^18–27^. It is known that nickel has a lower price, heightened reactivity, is more abundant, and can be used in many organic reactions^28–37^. The nickel-catalyzed Suzuki reaction has a mechanism similar to that of palladium^38^.

Environmental crises are increasing day-to-day with the fast development of modern industry. Greener and environmentally synthetic protocols and reaction conditions have crucial roles in the goal of green chemistry. Water is a common choice for green organic conversion, and its usage will have the slightest impact on nature. Water is an inexpensive, non-poisonous, available, green, non-flammable, eco-friendly, and excellent replacement for organic solvents. Therefore, scientists are interested in using catalysts in aqueous media owing to their benefits^39–42^.

Cyclodextrin (CD) compounds are an essential family of macrocyclic oligosaccharides with conical cavities and vast applications in various fields, such as the pharmaceutics and cosmetics industries^43–48^. It is well known that CD has encapsulation behavior, which shows the potential to create inclusion complexes connected with different guests. CDs have an inflexible cyclic configuration with a hydrophilic outer and a lipophilic cavity inner, revealing outstanding performances in water-mediated organic synthesis^49–56^. CDs with unique properties such as low toxicity, commercially available, water-soluble, biocompatibility, nontoxic, readily functionalized, and environmentally sound have lately obtained huge attraction as green catalysts in various organic reactions, especially for Suzuki–Miyaura coupling reactions^57–62^.

In recent years, following the latest papers, researchers are trying to design simple, green, inexpensive, and more efficient strategies for the Suzuki coupling^63–68^ due to environmental concerns and economic issues. So, to expand the attempts towards the Ni-catalyzed coupling reactions, we illustrate herein the coupling of aryl halide derivatives (X = Br and I) together with aryl boronic acid in aqueous conditions by using Ni(II)-β-CD catalytic.

## Results and discussion

**Figure 2:**
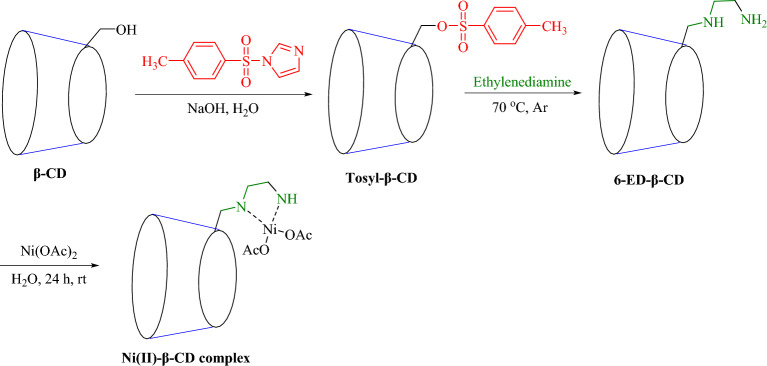
Schematic route for constructing Ni(II)-β-CD complex.

The provisioning procedure of Ni(II)-β-CD complex has four steps summarized in Fig. 2. First, commercial β-CD was treated with TsCl in two steps using a previous method^69^. In the next step, tosyl-β-CD was reacted with ethylene diamine at 70 °C to afford functionalized 6-ED-β-CD^70^. Then, the obtained ligand was stirred with Ni(OAc)_2_ in a water solution for 24 h, and the desired complex was obtained as a light green powder. Notably, the catalyst has displayed excellent solubility in the aqueous phase.

### Characterization

This Ni(II)-β-cyclodextrin complex was characterized by UV–Vis, AAS, TGA, and FT-IR techniques.

**Figure 3:**
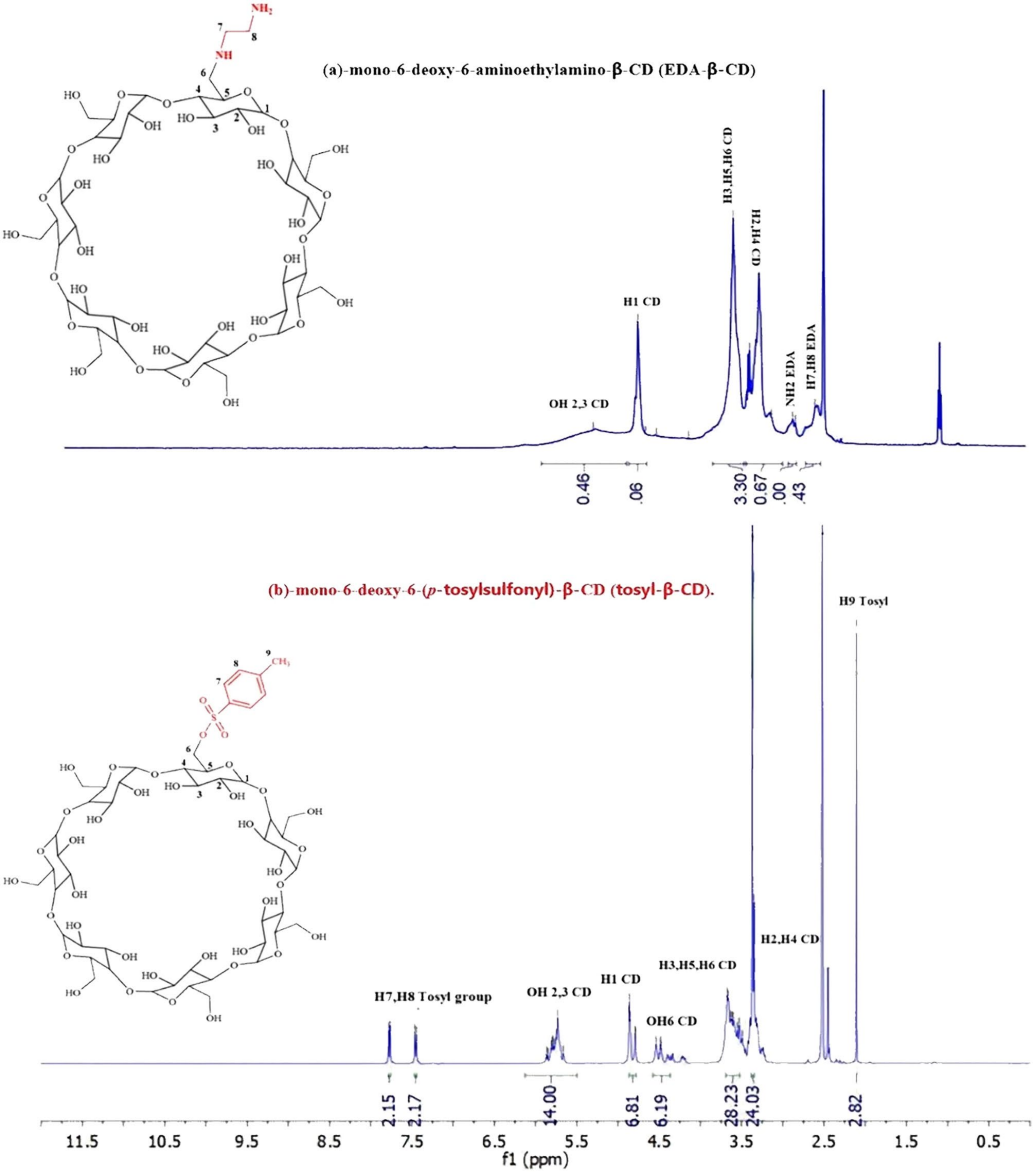
^1^H-NMR of mono-6-deoxy-6-(*p*-tosylsulfonyl)-β-CD (tosyl-β-CD) and mono-6-deoxy-6-aminoethylamino-β-CD (EDA-β-CD).

^1^H-NMR analysis of tosyl-β-CD and EDA-β-CD was investigated (Fig. 3). The emergence of the peaks related to the hydrogens of the methyl tosyl part in the area of 2.11 ppm and also the observation of the hydrogens of the aromatic range associated with the benzene tosyl ring in the areas of 7.45–7.79 ppm confirms the successful synthesis of tosyl-β-CD.

#### NMR analysis

In the ^1^H-NMR analysis of EDA-β-CD, the removal of the peaks associated with the tosyl ring in the 7.45–7.79 ppm regions and the appearance of the peak in the 2.63 ppm regions related to the four hydrogens of ethylenediamine indicates the successful synthesis of EDA-β-CD.

^1^H-NMR analysis cannot help to verify the complex construction of the Ni(II) with β-CD because nickel is paramagnetic, and ^1^H-NMR analysis cannot be confirmed. So, we use other analyses like FT-IR, TGA, and UV–Vis spectra.

#### TGA analysis

**Figure 4:**
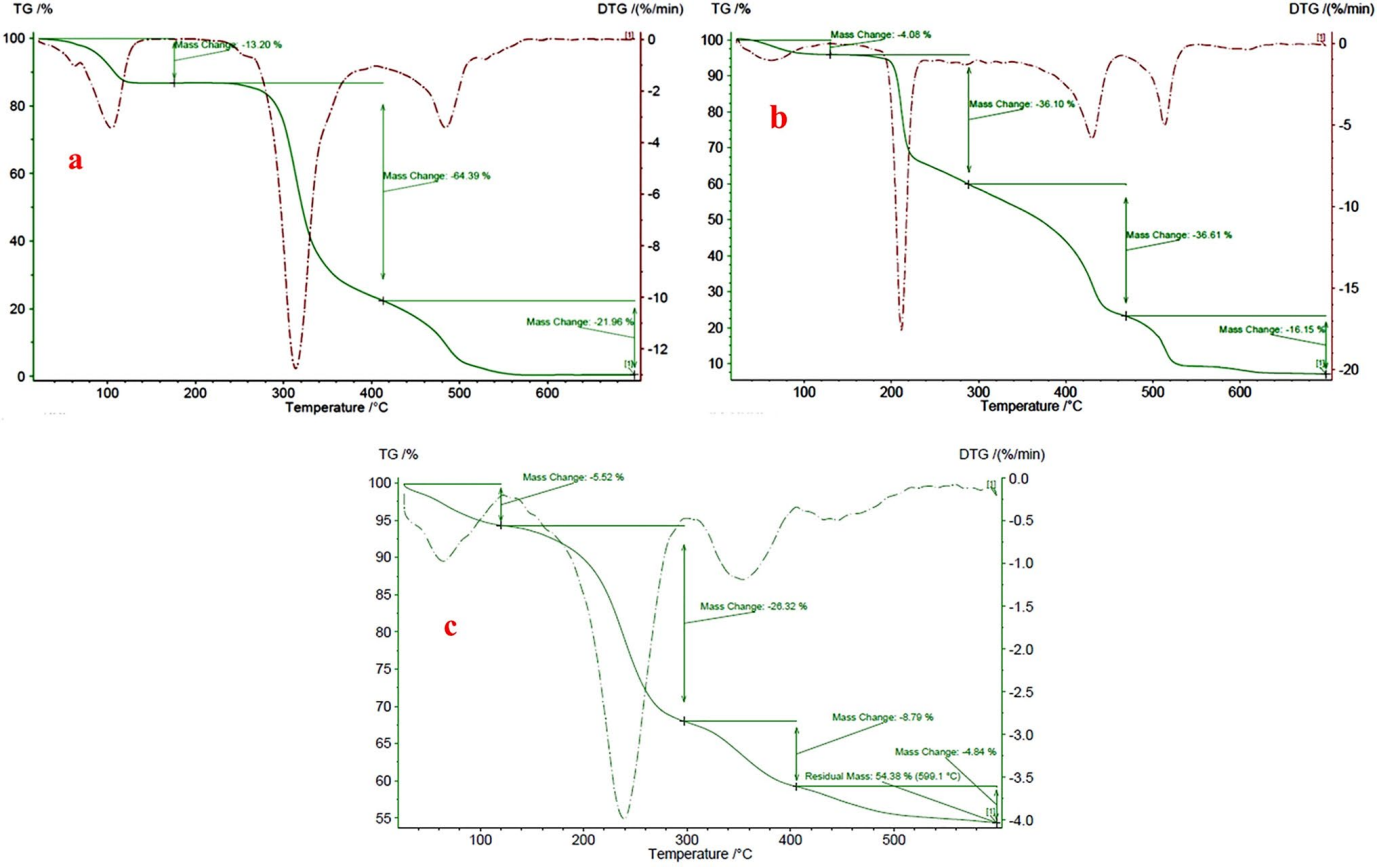
TGA curve of β-CD (**a**), EDA-β-CD (**b**), and Ni(II)-β-CD (**c**).

The thermal stability of this complex was surveyed by TGA analysis. Figure 4 shows the TGA spectrum of β-CD, ligand (EDA-β-CD), and Ni(II)-β-CD complex. The TGA curve of β-cyclodextrin (Fig. 4a) shows a weight loss of 13.20% below 150 °C, due to the loss of the adsorbed water. The mass loss of about 64.39% by weight in the 400–600 °C range is related to the thermal decomposition of β-cyclodextrin. The TGA curve of EDA-β-CD (Fig. 4b) displays a weight loss of 4.60% below 150 °C is due to the loss of the adsorbed water. The mass loss of about 36.10% by weight in the range of 300–450 °C is related to the thermal decomposition of the ethylenediamine. The last loss in the 480–600 °C range is attributed to β-CD. The TGA curve of the Ni(II)-β-CD complex (Fig. 4c) is entirely not similar to the TGA curve of β-CD and ligand; for the Ni(II)-β-CD complex, the TGA curve displays a weight drop of 5.52% in the scope of 25–120 °C, which is associated with physisorbed water and organic solvents. Additionally, around 26.32% of weight loss between 120 and 320 °C is related to the disintegration of complex Ni(II)-β-CD. This diagram confirms the thermal stability of the prepared catalyst. Also, nickel loading on the Ni(II)-β-CD complexes was 1.63 ppm, measured by atomic absorption spectroscopy (AAS).

#### UV–Vis analysis

**Figure 5:**
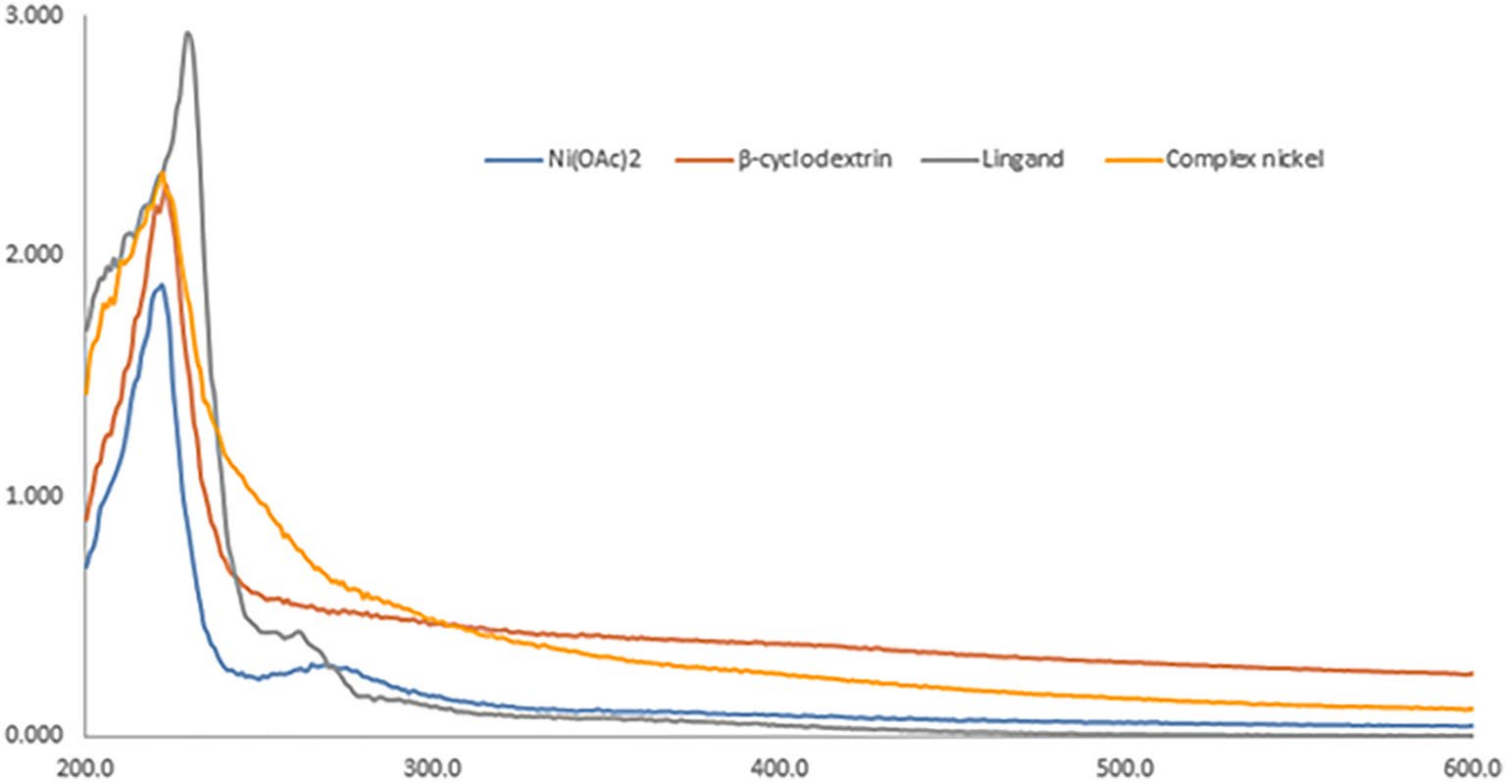
UV–vis spectroscopy of Ni(OAc)_2_ (blue), β-CD (orange), ligand (EDA-β-CD) (gray), and Ni(II)-β-CD complex (yellow).

UV–Vis spectra of the Ni(OAc)_2_ (blue), β-CD (orange), amino-β-CD (gray), and Ni(II)-β-CD complexes (yellow) were recorded from 200 to 600 nm in water (Fig. 5). Data showed that the corresponding bond at 240 nm related to nickel(II) acetate vanished in the existence of ligand (amino-β-CD) that conformed to the formed complex Ni(II)/β-CD.

#### FT‑IR analysis

**Figure 6:**
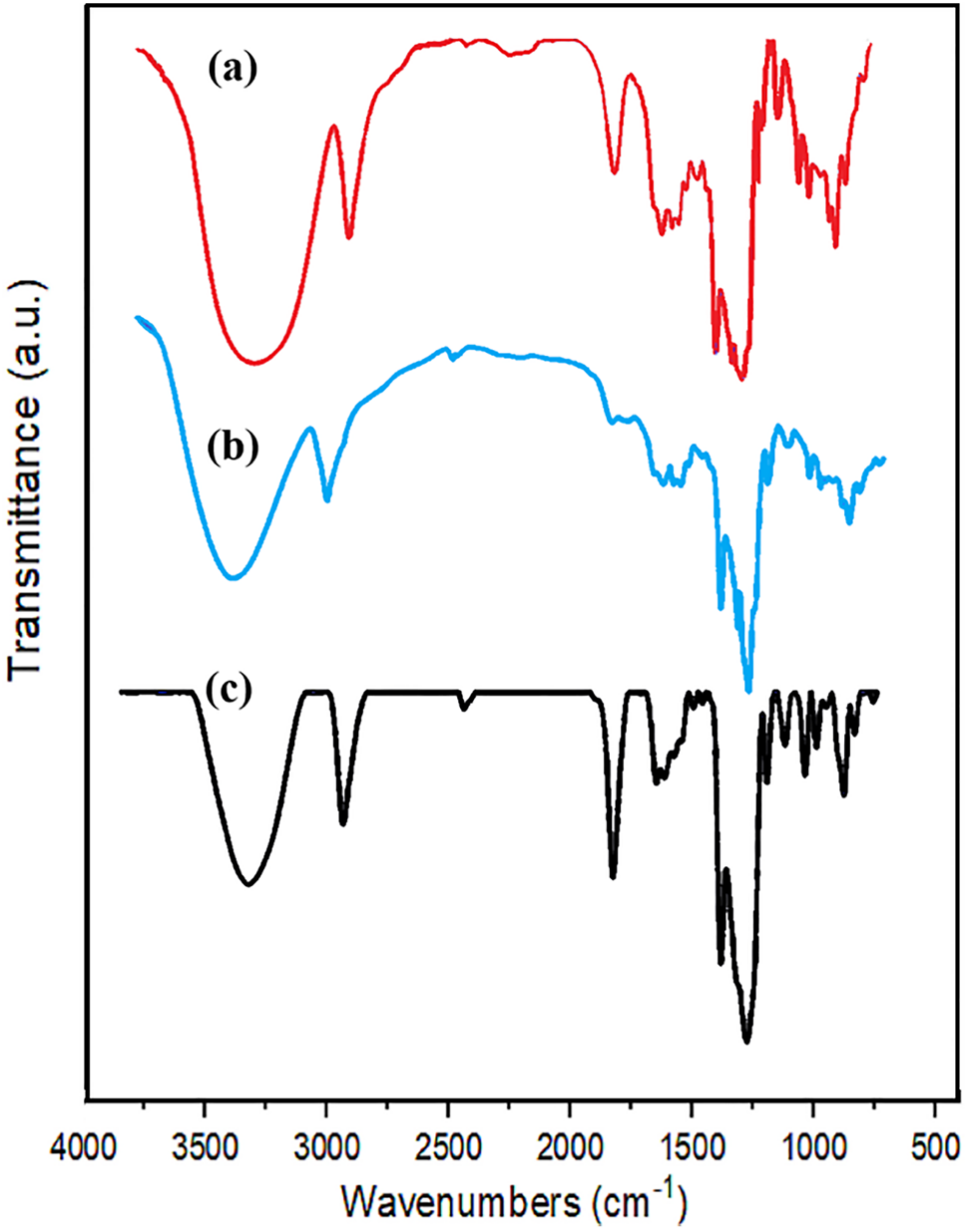
Infrared spectra spectra of β-CD (**a**), EDA-β-CD (**b**), and Ni(II)-β-CD (**c**).

Figure 6 includes the FT-IR spectra acquired for β-CD (curve a), EDA-β-CD (curve b), and Ni(II)-β-CD complex (curve c). FT-IR analysis of EDA-β-CD ligand showed wide bands at 1156 cm^−1^ (C–N), 2925 cm^−1^ (C–H), and 3347 cm^−1^ (O–H). The infrared spectrum of the Ni(II)-β-CD complex exhibits the distinct peaks of the hydroxyl group β-CD, which narrowed after being bonded with Ni. The existence of the C–O–C bond at 1033 cm^−1^ was shown. Also, the drastic band at 1661 cm^−1^ could be correlated to the deformation vibrations of H_2_O captured in the Ni(II)-β-CD complex^71^.

### Catalytic performance

The catalytic performance of the obtained supramolecular catalyst was assessed in the Suzuki reaction. To detect optimized status, the reaction of *p*-iodotoluene and benzeneboronic acid was chosen as the standard reaction, and the impact of varied parameters like temperature, base, solvent, and catalyst amount was examined. The outcome of the optimization experiments is listed in Tables 1 and 2. At the start, the effect of different transition metal salts was studied, and results showed that the reaction failed to obtain the expected product (Table 1, entries 1–7).

**Table 1:** Screening of different metal catalysts for reacting *p*-iodotoluene with benzeneboronic acid.


Entry	Metal catalysts	Solvent	Base	Yield^a^ (%)
1	Ni(NO_3_)_2_⋅6H_2_O	H_2_O	K_2_CO_3_	6
2	Ni(SO_4_)_2_⋅6H_2_O	H_2_O	K_2_CO_3_	3
3	NiCl_2_⋅6H_2_O	H_2_O	K_2_CO_3_	5
4	Ni(OAc)_2_	H_2_O	K_2_CO_3_	12
5	Co(OAc)_2_⋅6H_2_O	H_2_O	K_2_CO_3_	N.R
6	CoCl_2_⋅6H_2_O	H_2_O	K_2_CO_3_	N.R
7	FeCl_3_⋅6H_2_O	H_2_O	K_2_CO_3_	10
8	Ni-EDA-β-CD complex	H_2_O	K_2_CO_3_	79

Reaction status: phenylboronic acid, *p*-iodotoluene, base (with the ratio: 0.75:0.5:0.75), metal catalyst (5 mol%), and solvent (1 mL) undergo Argon.

^a^Yield determined by GC.

**Table 2:** Condition optimization for the reaction of *p*-iodotoluene and benzeneboronic acid-catalyzed using the Ni(II)-β-CD complex.


Entry	Catalyst (mol%Ni)	Base	Solvent	Yield^a^ (%)
1	2.5	K_2_CO_3_	THF	5
2	2.5	K_2_CO_3_	Toluene	7
3	2.5	K_2_CO_3_	CH_3_CN	11
4	2.5	K_2_CO_3_	dioxane	2
5	2.5	K_2_CO_3_	DMF	4
6	2.5	K_2_CO_3_	EtOH	35
7	2.5	K_2_CO_3_	MeOH	21
8	2.5	K_2_CO_3_	DMSO	Trace
**9**	**2.5**	**K**_**2**_**CO**_**3**_	**H**_**2**_**O**	**96**
10	2.5	K_2_CO_3_	H_2_O	41^b^
11	0.5	K_2_CO_3_	H_2_O	24
12	1	K_2_CO_3_	H_2_O	50
13	1.5	K_2_CO_3_	H_2_O	65
14	2	K_2_CO_3_	H_2_O	79
15	2.5	Cs_2_CO_3_	H_2_O	76
16	2.5	Na_2_CO_3_	H_2_O	79
17	2.5	DABCO	H_2_O	8
18	2.5	NEt_3_	H_2_O	Trace
19	2.5	t‐BuOK	H_2_O	55

Reaction status: phenylboronic acid, *p*-iodotoluene, base (with the ratio: 0.75:0.5:0.75), Ni(II)-β-CD complex, and solvent (1 mL) undergo Argon.

^a^Yield determined by GC.

^b^The reaction was conducted at ambient temperature.

Significant values are in bold.

Then, the reaction was studied under various solvents like THF, Toluene, CH_3_CN, dioxane, DMF, EtOH, MeOH, and DMSO by employing potassium carbonate as a base at 70 °C (Table 2, entries 1–8). The outcomes revealed an excellent yield (96%) using 2.5 mol% of Ni-EDA-β-CD complex in the water solvent, K_2_CO_3_, as a base for 18 h (Table 2, entry 9). Decreasing the temperature to rt reduced the yield to 41% (Table 2, entry 10). We used a smaller amount of Ni (0.5–2 mol%) at 70 °C (Table 2, entries 11–14), and the reaction yield was reduced under optimized conditions. Screening of various organic and inorganic bases Cs_2_CO_3_, Na_2_CO_3_, DABCO, Et_3_N, and t‐BuOK showed that the K_2_CO_3_ is most pleasing (Table 2, entries 15–19).

Having the optimized conditions, the domain of the Suzuki reaction was studied for different aryl halides with phenylboronic acids, and the obtained outcomes are listed in Table 3. As demonstrated in Table 3, the coupling reaction between phenylboronic and aryl iodides comprising electron-rich groups (Table 3, entries 1–3) as well as electron-poor groups (Table 3, entries 4–7) was carried out effectively to furnish the desired compounds in excellent yields. In addition, reactions of diverse aryl bromides involving the two electron-rich and electron-poor groups with phenylboronic acid progressed sufficiently, and favorable coupling products were gained in top yields (Table 3, entries 8–14).

**Table 3:** Ni(II)-β-CD as a catalyst in preparing coupling products.

Entry	Aryl halide	Product^a^	Time (h)	Yield^b^ (%)	M.p. (^o^C)^Ref^
1			5	97^c^	68–70^58^
2			6	95^c^	87–89^58^
3			6	94^c^	46–47^58^
4			6.5	85	70–72^58^
5			8	90	110–112^58^
6			8	91	84–86^58^
7			7.5	89	75–79^58^
8			8	90	68–70^58^
9			12	91	84–86^58^
10			12	87	110–112^58^
11			12	85	46–47^58^
12			12	86	87–89^58^
13			12	92	58–59^56^
14			12	90	119–120^56^

^a^Reaction status: aryl bromide/iodide, K_2_CO_3_, phenylboronic acid (with the ratio: 0.5:0.75:0.75), and water (1 mL) under an argon atmosphere.

^b^Isolated yields.

^c^Yields appointed by GC.

**Figure 7:**
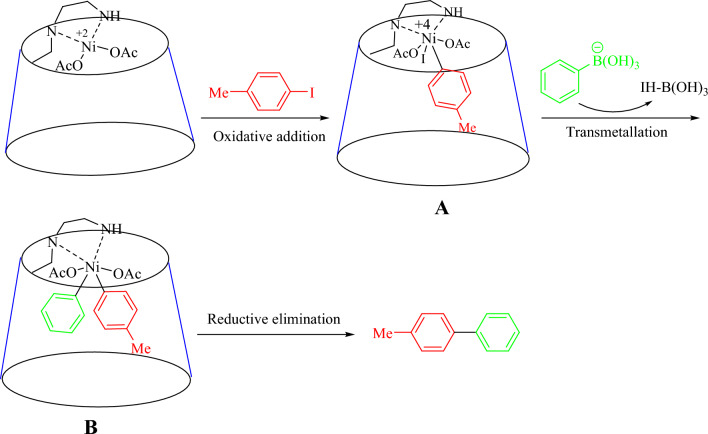
The proposed route for the Suzuki coupling using Ni(II)-β-CD.

Based on prior reports, a reasonable mechanism for the Suzuki reaction is exhibited in Fig. 7. Suzuki‐coupling mechanisms include three consecutive stages: oxidative addition, transmetalation, and reductive elimination. In the beginning step, Ni(II)-β-CD was initiated to Ni(0); afterward, the oxidative addition of aryl halides with active Ni(II)-β-CD led to the production of intermediate A. In continuation, the insertion of the phenyl from benzeneboronic acid via the transmetalation reaction obtained intermediate B. In the final step, the coupling product was removed from the cavity, and reductive elimination of the Ni(0) species.

### Comparison of catalytic activity

To avoid using perilous metals in the Suzuki coupling, boronic acid, and aryl halide are increasingly essential to develop low-cost metal transition metal catalysts like nickel. Nickel catalysts are well-known for their promising highly catalytic activity in the Suzuki reaction and as a low-cost alternative for palladium in cross-coupling reactions. A list of Nickel-catalyzed Suzuki reactions is shown in Table 4, and comparison results about the catalytic activity of Ni(II)-β-CD with several other catalysts Suzuki reaction indicated high catalytic efficiency of the former catalyst. This catalyst has some advantages compared to past works, such as using water as a solvent, while in most of the prior reports, dioxane, toluene, DMSO, and PEG-400 solvents were applied. The reaction conditions were carried out at high temperatures in the prior works, whereas in the present work, at an optimal temperature of 70 °C. Our method is cost-effective as Ni-β-CD is cheaper than other nickel catalyst complexes used in past reports.

**Table 4:** The comparative catalytic performance of the Ni(II)-β-CD complex with other reported Ni catalysts in the Suzuki coupling.

Entry	Catalyst	Temp. (°C)	Time (h)	Solvent	Ni (mol%)	Yield (%)	Ref
1	Cp2Ni/Xantphos	130	16	Toluene	5	17–97	63
2	[Ni(NHC)BrCp]	110	1–2	Toluene	1–3	52–90	64
3	Ni(II)-a-diimine-PO	100	8–12	Toluene	5	90–99	65
4	NiCl_2_(dppp)	100	8–24	PEG-400	2	71–95	66
5	GO/NiTAPP	80	1–2.5	Dioxane	3	60–95	67
6	NiCl_2_.glyme	80	12	DMSO/Glycerol	10	71–90	68
7	Ni(OAc)_2_/β-CD	65–85	24	H_2_O	5	82–98	62
**8**	**Nickel complex with functionalized amino-β-CD**	**70**	**5–12**	**H**_**2**_**O**	**2.5**	**85**–**97**	**This work**

Significant values are in bold.

## Experimental

### Chemicals and instruments

Nickel(II) acetate (Ni(OAc)_2_), potassium carbonate (K_2_CO_3_), aryl halides (ArX), aryl boronic acids (ArB(OH)_2_), ethylenediamine, *p*-toluenesulfonyl chloride, imidazole, and β-CD were provided from Sigma. TLC kept the reaction under observation, and GC analysis was carried out on the Varian CP 3800 chromatograph. The ^1^H-NMR spectra of compounds were achieved using DRX-400 from Bruker company in DMSO-*d*_*6*_ or CDCl_3_. Thermogravimetric analysis (TGA) was obtained on the NETZSCHSTA 409 PC/PG instrument. The nickel content of the synthesized catalysts was specified by Varian spectrum110 atomic absorption spectrometry. FT-IR studies were performed on a Bruker Vector 22 FT-IR spectrophotometer using a KBr pellet. The UV–Vis spectra were assayed using a UV–Vis spectrophotometer (JASCO, UV-550).

### Preparation of mono-6-tosyl-β-CD (Tosyl-β-CD)

Mono-6-O-*p*-toluenesulfonyl-β-CD (Tosyl-β-CD) was obtained as mentioned in the former methods^69^. β-CD (4.4 mmol, 5.0 g) and tosyl imidazole (13.5 mmol, 3 g) were dissolved in H_2_O (125 mL) under vigorous mechanical stirring. Afterward, after 6 h, NaOH (56.25 mmol, 2.25 g) in H_2_O (6.52 mL) was added at a leisurely pace for 20 min. The mixture was quenched by adding NH_4_Cl (112.5 mmol, 6.03 g). Precipitating the tosyl-β-CD (14.1%) was obtained from the solution by blowing air onto its surface for 24 h and rinsing it twice with acetone and ice water.

### Preparation of mono-6-deoxy-6-aminoethylamino-β-CD (EDA-β-CD)

6-OTs-β-CD (1.16 mmol, 1.50 g) dissolved in ethylenediamine (10 mL) was stirred at reflux condition at 70 °C under an N_2_ atmosphere (14 h)^70^. The unreacted ethylenediamine was eliminated via rotary evaporation, and the remains precipitated into cold acetone. The residue was rinsed in acetone and H_2_O and dried. (yield: 66%, white powder).

### Preparation of Ni(II) complex of amino-β-CD

To a flask including amino-β-CD (500 mg, 0.4 mmol) and Ni(OAc)_2_ (0.8 mmol, 141 mg), H_2_O (5 mL) was added. Then, the mixture was stirred at 25 °C for one day. Then, acetone was added to this solution until a light green powder precipitate. Precipitated Ni(II)-β-CD complex was filtrated and rinsed with acetone to eliminate free nickel dried in an oven (60 °C).

### General procedure for the Suzuki coupling

The catalyst (288 mg, 2.5 mol%) was added to a combination of aryl halide (0.5 mmol), phenylboronic acid (0.75 mmol), and K_2_CO_3_ (0.75 mmol) in H_2_O (1 mL), and the mixture was stirred at 70 °C (18 h) under argon atmosphere. TLC and GC followed the progress. Following finishing the reaction, the crude product was elicited using n-hexane. The desired products were obtained by column chromatography on silica using ethyl acetate and hexane as eluent. The spectroscopic data of the final products are included in supplementary information.

### Catalyst reusability

**Figure 8:**
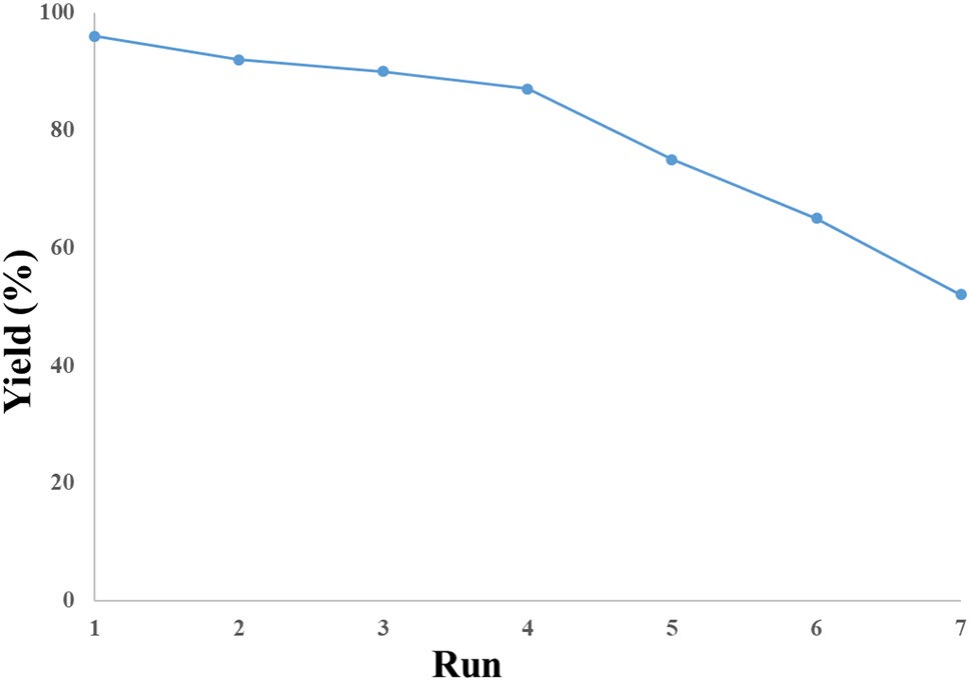
Recycling outcomes for the Suzuki reaction of benzene boronic acid with *p*-iodotoluene.

The catalyst recycling was investigated in coupling p-iodotoluene and benzene boronic acid under the optimized status. After the reaction, the aqueous solution comprising the catalyst was moved to a new flask and applied in another batch. This procedure was done for seven sequential runs, and minute drops in its activity were observed. The results exhibited that the GC yield of the reaction reduced from 96 to 90% in batch 3, and from batch 3 to 6, the yield decreased to 65%. However, in run 7, the reaction yield was competently lowered to 52%. (Fig. 8).

**Figure 9:**
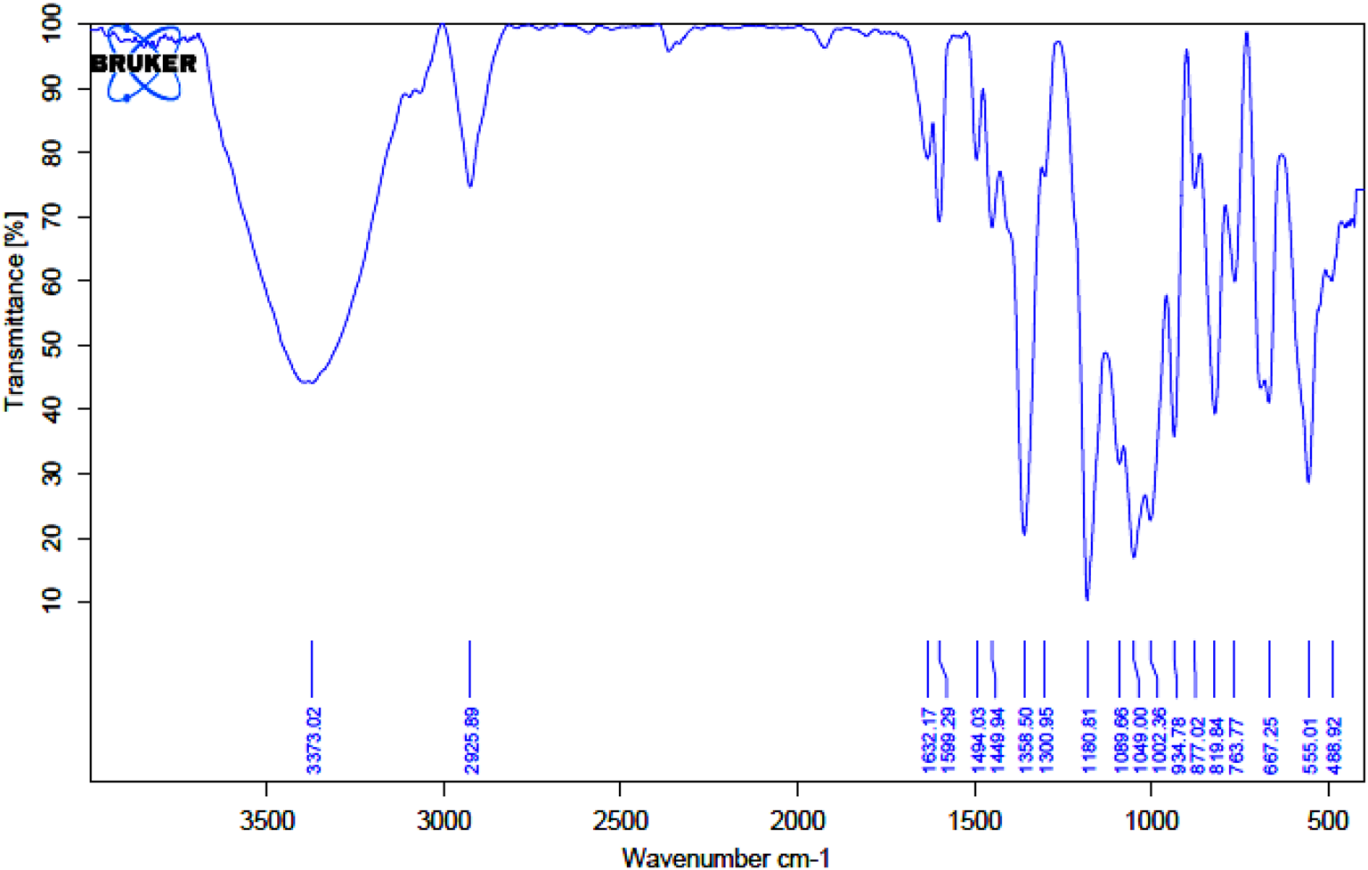
FT-IR was acquired for the reused catalyst after the four runs from the Suzuki reaction.

**Figure 10:**
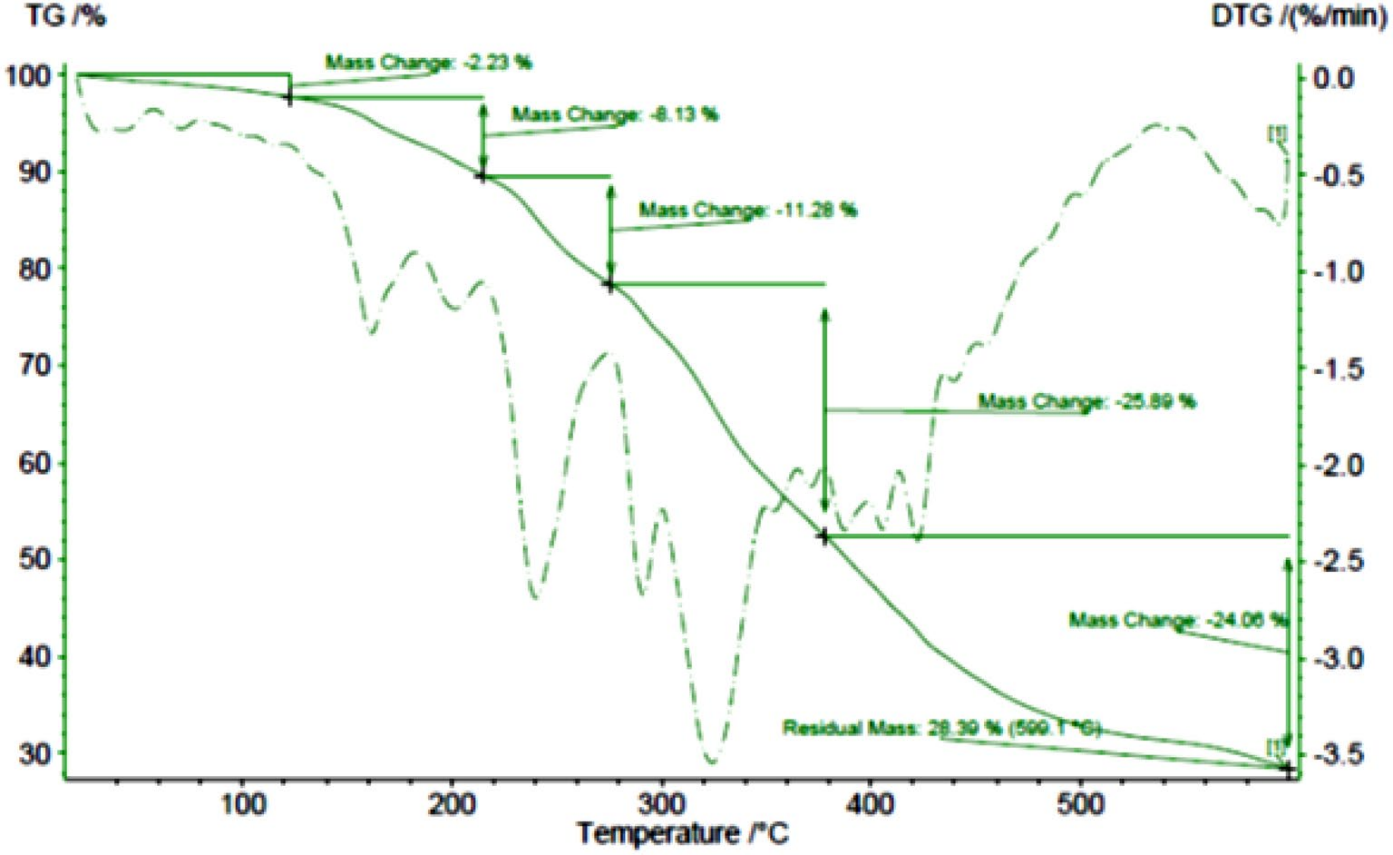
TGA plot of the catalyst after the four times from the Suzuki reaction.

FT-IR (Fig. 9) and TGA (Fig. 10) images of the catalyst after the four times exhibited that the structure of the catalyst was conserved.

### Hot filtration

**Figure 11:**
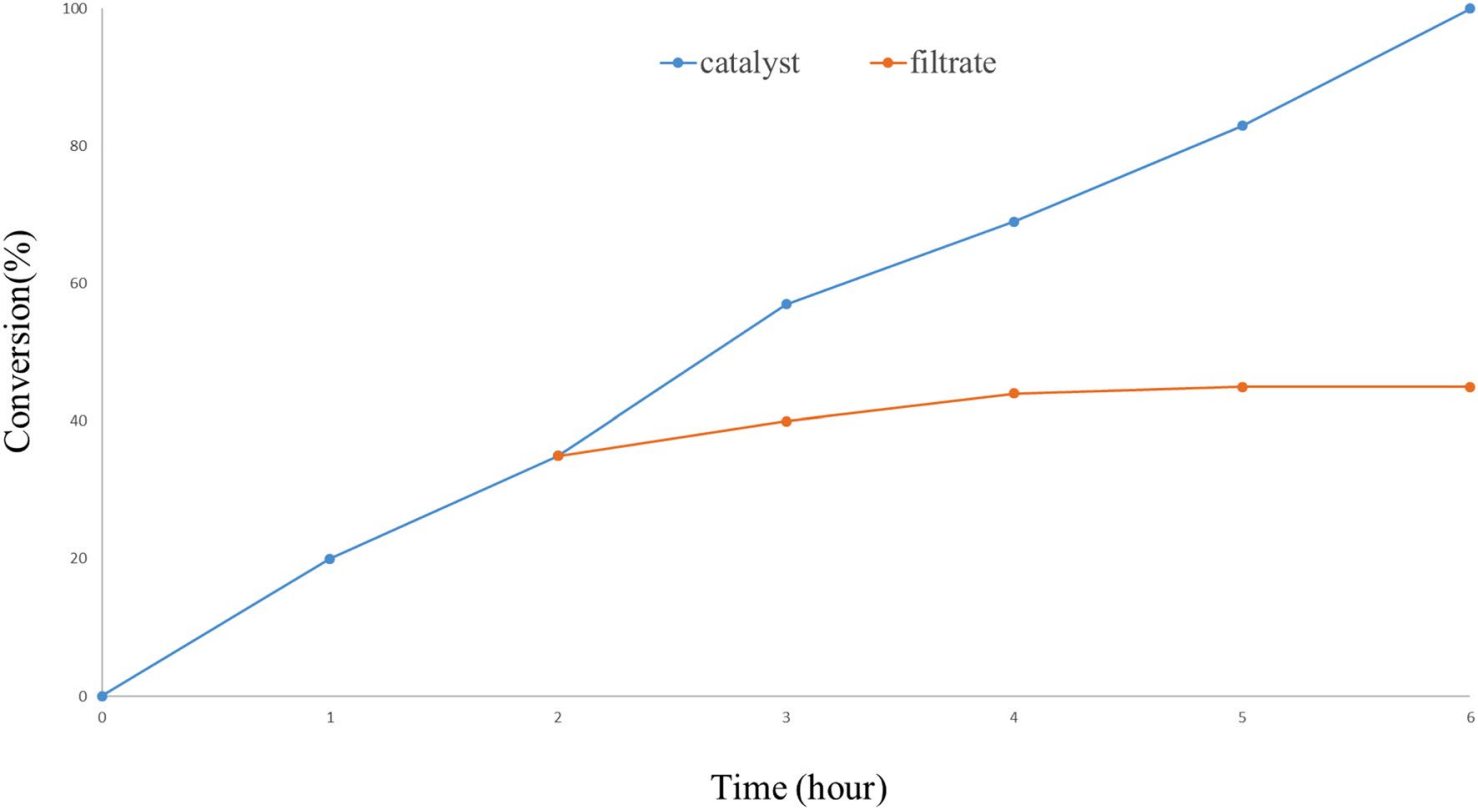
Hot filtration test for Ni(II)-β-CD complex.

Hot filtration was performed for the *p*-iodotoluene and benzeneboronic acid reaction under the optimized reaction status (Fig. 11). At the time of the test, the catalyst was stirred in water for 30 min (70 °C). Afterward, the catalyst was eliminated by filtration, and *p*-iodotoluene, potassium carbonate, and phenylboronic acid were added. After 2 h, GC analysis of the reaction indicated the construction of 35% of the expected coupling product and 65% of *p*-Iodotoluene. The result showed that after 2 h, the reaction progressed slowly, and the coupling product created a 45% GC yield.

## Conclusion

We have designated a water-soluble, readily available, greener, and cheap nickel catalyst to synthesize biaryl compounds for the Suzuki coupling reaction. β-CD was a green and cheap source of natural cyclic oligosaccharides with excellent properties like formation inclusion complexes with various metals. In the present study, we have introduced β-CD Ni(II)complex as a green catalyst for the carbon–carbon bond construction through Suzuki coupling of various organic aryl halides and benzeneboronic acid in neat water to biaryl products obtained in excellent to high yield. The ligand is readily synthesized in three steps and then combined with Ni(OAc)_2_ to make an economical catalyst utilized in the Suzuki reaction. This supramolecular homogeneous catalyst was recycled for four runs with preservation of the catalytic activity in Suzuki reactions.

### Supplementary Information

Supplementary Information.

## Data Availability

All data have been given in the article and supporting information.
