# Supplementary material for: A new β-cyclodextrin-based nickel as green and water-soluble supramolecular catalysts for aqueous Suzuki reaction
Source: Sci Rep. 2023 Dec 2;13:21279. doi: 10.1038/s41598-023-48603-6 (PMC10693598; doi:10.1038/s41598-023-48603-6)
Supplement: Supplementary file 1 — Supplementary Information. [file 41598_2023_48603_MOESM1_ESM.pdf]

Supporting information

A new β-cyclodextrin-based nickel as green and water-soluble supramolecular catalysts for aqueous Suzuki reaction

Sara Payamifar and Ahmad Poursattar Marjani*

Department of Organic Chemistry, Faculty of Chemistry, Urmia University, Urmia, Iran,

*E-mail: a.poursattar@urmia.ac.ir; a.poursattar@gmail.com

^{1}H and ^{13}C- NMR of products

The NMR spectrum synthesized compounds are known compounds[1].

Biphenyl

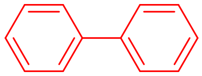

White solid; mp: 68-70 °C. ^{1}H-NMR ($CDCl_3$, 400 MHz) δ (ppm): 7.71–7.69 (m, 2H), 7.56–7.52 (m, 2H), 7.45–7.43 (m, 1H). ^{13}C-NMR (100 MHz, $CDCl_3$) δ (ppm): 141.32, 128.86, 127.44, 127.27.

4-Methoxy biphenyl

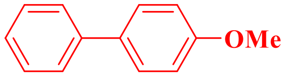

White solid; mp: 87–89 °C. ^{1}H NMR ($CDCl_3$, 400 MHz) δ (ppm): 7.60 (t, *J* = 8.4 Hz, 4H), 7.47 (t, *J* = 7.6 Hz, 2H), 7.34 (d, *J* = 7.3 Hz, 1H), 7.04 (d, *J* = 8.7 Hz, 2H), 3.11(s, 3H). ^{13}C-NMR (100 MHz, $CDCl_3$) δ (ppm): 159.29, 140.77, 133.74, 128.76, 128.20, 126.78, 126.70, 114.47, 55.38.

4-Methyl biphenyl

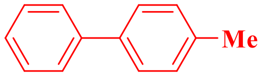

White solid; mp: 46–47 °C. ^{1}H-NMR ($CDCl_3$, 400 MHz) δ (ppm): 7.68–7.62 (m, 2H), 7.56 (d, J = 8.1 Hz, 2H), 7.49 (t, J = 7.6 Hz, 2H), 7.39 (t, J = 7.4 Hz, 1H), 7.34 (d, J =7.9 Hz, 2H), 2.50 (s, 3H). ^{13}C-NMR (100 MHz, $CDCl_3$) δ (ppm): 141.16, 138.48, 137.14, 129.53, 128.76, 127.23, 127.03, 21.15.

4-Chloro-1,1'-biphenyl

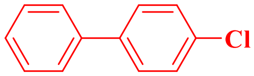

White solid; mp: 70–72 °C.^{1}H-NMR ($CDCl_3$, 400 MHz): δ (ppm): 7.65–7.59 (m, 4H), 7.55–7.45 (m, 5H). ^{13}C-NMR ($CDCl_3$, 100 MHz) δ (ppm): 140.03, 139.71, 133.43, 128.98, 128.96, 128.46, 127.66, 127.05.

4-Nitro-1,1'-biphenyl

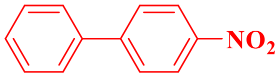

Yellowish solid; mp: 110–112 °C. ^{1}H-NMR (400 MHz, $CDCl_3$) δ (ppm): 8.33–8.35 (m, 2H), 7.77–7.79 (m, 2H), 7.68–7.66 (m, 2H), 7.56–7.47 (m, 3H). ^{13}C-NMR (100 MHz, $CDCl_3$) δ (ppm): 147.66, 147.11, 138.85, 129.18, 128.94, 127.83, 127.41, 124.14.

***p*-Benzonitrile-biphenyl**

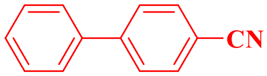

White solid; mp: 84–86 °C. ^{1}H-NMR ($CDCl_3$, 400 MHz) δ (ppm): 7.74 (q, J = 8.5 Hz, 4H), 7.66–7.61 (m, 2H), 7.57–7.43 (m, 3H). ^{13}C-NMR (100 MHz, $CDCl_3$) δ (ppm): 145.75, 139.41, 132.63, 129.25, 128.69, 127.77, 127.26, 119.03, 110.93.

4-Fluoro-1,1'-biphenyl

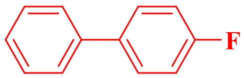

White solid; mp: 75–79 °C. ^{1}H-NMR ($CDCl_3$, 400 MHz) δ (ppm): 7.63–7.56 (m, 4H), 7.50–7.47 (m, 2H), 7.42–7.38 (m, 1H), 7.22–7.13 (m, 2H). ^{13}C-NMR (100 MHz, $CDCl_3$) δ (ppm): 163.86, 161.16, 140.49, 137.40, 128.86, 128.77, 128.69, 127.30, 127.06, 115.76, 115.50.

***p*-biphenyl benzaldehyde**

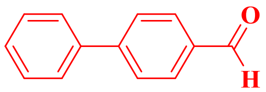

White solid; mp: 57–59 °C. ^{1}H-NMR ($CDCl_3$, 400 MHz) δ (ppm): 10.13 (s, 1H), 8.02–7.95 (m, 2H), 7.81–7.79 (m, 2H), 7.69–7.67 (m, 2H), 7.57–7.42 (m, 3H). ^{13}C-NMR (100 MHz, $CDCl_3$) δ (ppm): 192.05, 147.23, 139.74, 135.70, 132.76, 130.36, 129.09, 128.56, 127.74, 127.43.

Biphenyl-4-acetophenone

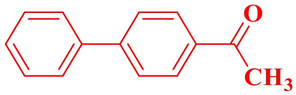

White solid; mp: 119–121 °C. ^{1}H-NMR ($CDCl_3$, 400 MHz) δ (ppm): 8.07 (d, J = 8 Hz, 2H), 7.76–7.70 (m, 2H), 7.66 (d, J = 7.5 Hz, 2H), 7.51 (t, J = 7.2 Hz, 2H), 7.45 (d, J = 6.5 Hz, 1H), 2.67(s, 3H). ^{13}C-NMR (100 MHz, $CDCl_3$): δ 197.79, 145.82, 139.91, 136.01, 128.99, 128.95, 128.27, 127.30, 127.26, 26.60.

Reference

1. (a) Bhojane, J. M., Sarode, S. A. & Nagarkar, J. M. *New J. Chem.* **40**, 1564–1570 (2016); (b) Liao, Y., Yang, W., Wei, T. & Cai, M. *Synth. Commun.* **49**, 1134–1142 (2019).
